# Construction of a Redox-Related Prognostic Model with Predictive Value in Survival and Therapeutic Response for Patients with Lung Adenocarcinoma

**DOI:** 10.1155/2022/7651758

**Published:** 2022-02-25

**Authors:** Lingyan Xiao, Qian Li, Yongbiao Huang, Zhijie Fan, Li Ma, Bo Liu, Xianglin Yuan

**Affiliations:** ^1^Department of Oncology, Tongji Hospital, Tongji Medical College, Huazhong University of Science and Technology, Wuhan, China; ^2^Department of Pathophysiology, School of Basic Medicine, Tongji Medical College, Huazhong University of Science and Technology, Wuhan, China

## Abstract

**Background:**

Lung adenocarcinoma (LUAD) represents the most common histological subtype of lung cancer. Redox plays a significant role in oncogenesis and antitumor immunity. In this study, we aimed to investigate the prognostic redox-associated genes and construct a redox-based prognostic signature for LUAD.

**Materials and Methods:**

A discovery cohort containing 479 LUAD samples from The Cancer Genome Atlas (TCGA) was analyzed. We identified prognostic redox-associated genes by weighted correlation network analysis (WGCNA) and univariate Cox regression analysis to construct a prognostic model via least absolute shrinkage and selection operator (LASSO)-multivariate Cox regression analyses. The performance of the redox-based model was validated in the TCGA cohort and an independent cohort of 456 samples by Cox regression analyses, log-rank test, and receiver operating characteristic (ROC) curves. Correlations of the model with clinicopathological variables and lymphocyte infiltration were assessed. Gene set enrichment analysis (GSEA) was used to clarify the underlying mechanism of the prognostic model. We constructed a nomogram based on the model and created calibration curves to show the accordance between actual survival and predicted survival of the nomogram.

**Results:**

Stepwise analyses identified 6 prognostic redox-associated genes of LUAD and constructed a prognostic model that performed well in both the discovery and validation cohorts. The model was found to be associated with tumor stage, mutation of TP53 and EGFR, and lymphocyte infiltration. The model was mainly involved in the regulation of the cell cycle, DNA replication and repair, NADH metabolism, and the p53 signaling pathway. Calibration curves showed the high predictive accuracy of the nomogram.

**Conclusions:**

This study explored the role of redox-associated genes in LUAD and constructed a prognostic model of LUAD. The signature was also associated with tumor progression and therapeutic response to immunotherapy. These findings contributed to uncovering the underlying mechanism and discovering novel prognostic predictor of LUAD.

## 1. Introduction

Comprising 40% of lung cancer cases, lung adenocarcinoma has occupied a core position in lung cancer due to its high mortality and morbidity. Although the 5-year survival rate has reached approximately 60% for patients with early-stage LUAD, the number declines to 25% when all stages are combined [1].

The prediction of survival for patients with LUAD can aid in tailoring optimal treatment strategies. The TNM staging system remains the most frequently used indicator to predict outcome for patients with LUAD. Targeted therapy and immunotherapy represent emerging and effective therapies for LUAD. For patients in the advanced stage without targeted mutations, immune checkpoint inhibitor (ICI) therapy may demonstrate satisfactory efficacy. With the rise of various treatment strategies, more reliable prognostic biomarkers are needed to achieve individual therapy.

Reactive oxygen species (ROS) are oxygen (O)-containing molecules generated mainly by oxidative phosphorylation in mitochondria and NADPH oxidases (NOXs). ROS are involved in various biological processes due to their high reactivity toward proteins, nucleic acids, and lipids [2]. ROS can affect the function of proteins by modifying cysteine residues, giving rise to diseases such as asthma, diabetes, and cancer [3–6]. Redox homeostasis refers to the balance between the generation and elimination of ROS [7]. Upsetting this delicate redox balance was related to the initiation and progression of cancer [8–10]. Oncogenesis and metastasis can be inhibited by high ROS concentration, and cancer cells can promote the synthesis of antioxidants to buffer ROS to avoid this inhibition [11]. Pathways that are essential to oncogenesis, such as PI3K/AKT, MAPK, and NF-*κ*B, are regulated by redox [12–15]. Oncogenes and tumor suppressor genes such as TP53, PTEN, and RAS can also interact with the redox system to determine the fate of cells [2, 16–20]. Cancer stem cells (CSCs) have been reported to have unique redox profiles with low ROS level facilitating the stemness properties and drug resistance of CSCs, and the ability of cancer stem cells to generate tumorspheres can be limited by ROS [21].

Given the great significance of redox in tumorigenesis, we hypothesized that redox-associated genes may have prognostic value in LUAD. We conducted multiple analyses to identify the significant prognostic redox-associated genes based on expression data of LUAD acquired from TCGA. A prognostic model was constructed based on prognostic redox-associated genes by LASSO and multivariate Cox regression analyses, which could predict the survival outcome and immunophenotypes of patients with LUAD.

## 2. Materials and Methods

### 2.1. Data Collection and Differential Expression Analysis

Expression data, mutation data, and corresponding clinical information of LUAD were downloaded from the TCGA database. A total of 594 samples were analyzed (N (normal) = 59, N (tumor) = 535), among which 522 samples had clinical information. Microarray data used for validation were acquired from the Gene Expression Omnibus (GEO) database (GSE32863, GSE43458, GSE37745, GSE31210, and GSE50081). A total of 4338 redox-associated genes were collected from the GSEA website (https://www.gsea-msigdb.org), the Gene Ontology website (http://geneontology.org/), OMIM database (https://omim.org/), and GeneCard database (https://www.genecards.org/). The package “edgeR” was used to remove the genes with low expression, normalize the expression data, and identify differentially expressed redox-associated genes. First, we conducted differential expression analysis between the total tumor and normal samples (*N* = 594). Next, the differential expression analysis between the paired tumor and peritumoral tissues (*N* = 114) was performed to eliminate the effect caused by the huge difference in the total sample number between the tumor and normal tissues. Genes meeting the filtering criteria of false discovery rate (FDR) < 0.05 and |log2 fold change (FC)| > 2.0 in two differential analyses were considered differentially expressed genes.

### 2.2. GO and KEGG Analyses

Gene Ontology (GO) enrichment and Kyoto Encyclopedia of Genes and Genomes (KEGG) pathway analyses were conducted using the *R* package “clusterProfiler” to clarify the functions of the dysregulated redox-associated genes. Selection criteria are both q value and *p* value < 0.05.

### 2.3. Establishing a Prognostic Model

WGCNA is a powerful bioinformatic tool for identifying co-expressed genes with similar biological functions to generate co-expression modules and correlating co-expression modules with parameters of interest [22]. Thus, WGCNA has been frequently applied in identifying biomarkers that are associated with specific biological functions or clinical characteristics [23–25]. In our study, WGCNA was used to extract significant gene modules associated with survival and clinical variables, including age, gender, and stage, using the “WGCNA” package. Next, univariate Cox regression analysis and log-rank test were performed for genes in the survival-related gene module to further identify prognostic redox-associated genes. LASSO and multivariate Cox regression analyses were conducted using the “glmnet” package to determine the prognostic genes for model construction, and the coefficients obtained in multivariate Cox regression were set as weights.

### 2.4. Verification of the Prognostic Model

Differential expression of the prognostic redox-associated genes in the model was verified in GSM43458 (*N* = 110) and GSM32863 (*N* = 116) using the “limma” package. Survival curves of these genes were obtained from Gene Expression Profiling Interactive Analysis (GEPIA) (http://gepia.cancer-pku.cn/). After removing the samples without complete survival and clinical data, GSE37745 (*N* = 196), GSE31210 (*N* = 79), and GSE50081 (*N* = 181) were merged into the GEO cohort (*N* = 456). The prognostic performance of the model was assessed in the TCGA and GEO cohorts. The Kaplan–Meier survival curves were plotted to compare the overall survival (OS) of the high- and low-risk groups. The correlation of the prognostic model with survival was indicated by the univariate Cox regression analysis. Then, the multivariate Cox regression analysis was performed to evaluate whether the risk score could affect the survival of patients with LUAD independently. The “survival” package was used to conduct the survival analyses. The predictive power of the prognostic model was assessed by ROC curves using the “survivalROC” package.

### 2.5. Relationship between the Prognostic Model and Clinical Parameters and Gene Set Enrichment Analysis

The distribution of the risk score in patients divided by clinical variables was compared to show the correlation between risk score and cancer progression. The mutation rate of driver genes was compared between the high- and low-risk groups based on the chi-square test. GSEA was performed to reveal the involved GO terms and pathways of our prognostic model based on filter criteria of NOM *p* value < 0.01 and FDR q value < 0.05.

### 2.6. Association of the Prognostic Model With Infiltration of Lymphocytes

Estimation of STromal and Immune cells in MAlignant Tumours using Expression data (ESTIMATE), an algorithm that can work out the proportion of stromal cells and immune cells [26], was applied to obtain the immune score and stromal score, which represented the abundance of immune cells and stromal cells, respectively. Major immune cells in the tumor microenvironment of the TCGA cohort were downloaded from TIMER [27, 28] (http://timer.comp-genomics.org). Cell-type Identification By Estimating Relative Subsets Of RNA Transcripts (CIBERSORT) is a computational method that uses a deconvolution algorithm to qualify the composition of cells based on expression data. Here, the CIBERSORT algorithm was run by *R* language to further show the proportion of tumor-infiltrating lymphocyte subsets [29]. The number of permutations was set to 1000.

### 2.7. Construction and Validation of a Nomogram

A nomogram based on the prognostic model was established using the “rms” package to estimate the survival probability of patients. To show the concordance between predicted survival and actual survival, the calibration curves were created for both the TCGA and GEO cohorts.

### 2.8. Statistical Analysis

All statistical analyses were carried out with *R* software 4.0.0. GSEA was conducted by the GSEA software version 4.1.0. The *p* value was corrected using the Benjamini–Hochberg approach in differential expression analysis between the normal and tumor tissues. Survival differences were evaluated by the log‐rank test. Differences in gene expression and immunophenotypes between the high- and low-risk groups were assessed by the Wilcoxon test, and gene mutation rates between the two groups were compared by the chi-square test. *P* < 0.05 was considered statistically significant.

## 3. Results

### 3.1. Differentially Expressed Redox-Associated Genes of LUAD

**Figure 1:**
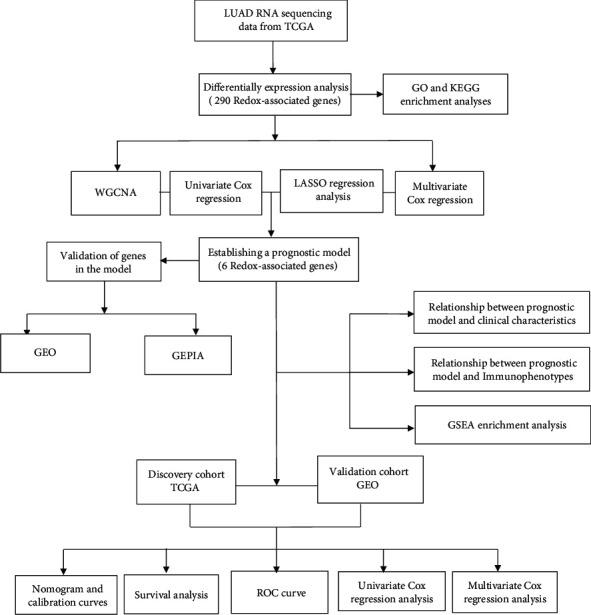
Flowchart of this study.

**Figure 2:**
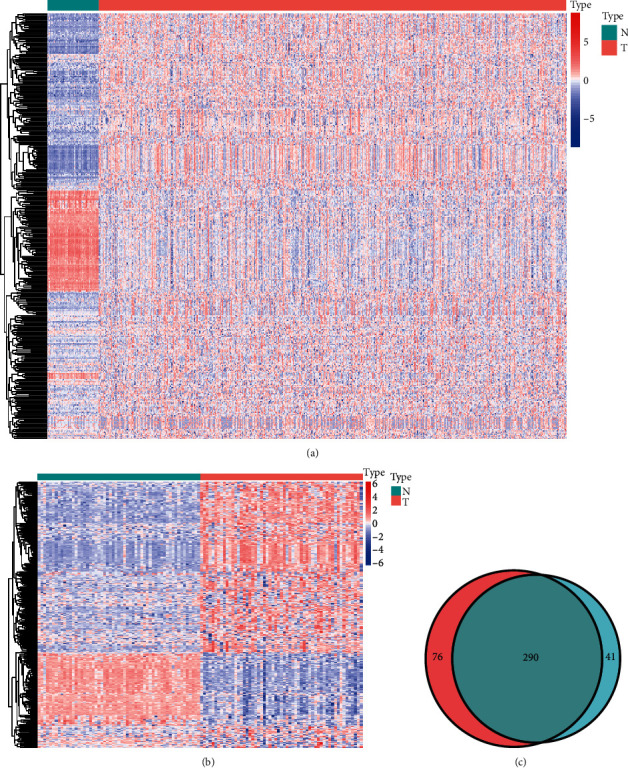
Differential expression analyses of redox-associated genes. (a) Heat map of differentially expressed redox-associated genes between overall lung adenocarcinoma tissues and normal lung tissues. (b) Heat map of differentially expressed redox-associated genes between paired lung adenocarcinoma tissues and peritumoral lung tissues. (c) Venn diagram of overlapping redox-associated genes in the two differential expression analyses.

Figure 1 presents the flowchart of our research. A total of 366 and 331 dysregulated redox-associated genes meeting the cutoff value of |log2 fold change (FC)| >2.0 and adjusted *p* value (FDR) < 0.05 were identified in the differential expression analysis of overall samples and paired samples, respectively (Figures 2(a) and 2(b)). A total of 290 overlapping dysregulated redox-associated genes were identified from these two differential expression analyses, including 75 downregulated genes and 215 upregulated genes (Figure 2(c)).

### 3.2. Functions of Dysregulated Redox-Associated Genes

**Figure 3:**
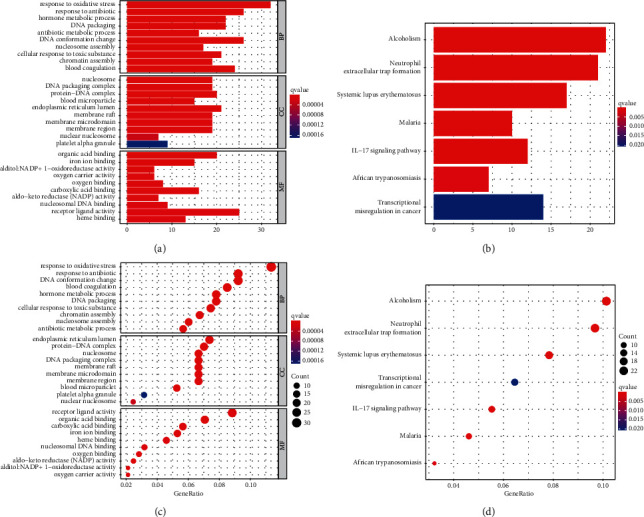
Involved GO terms and pathways of the dysregulated redox-associated genes. (a) Bar plots for the involved GO terms of the dysregulated redox-associated genes. (b) Bar plots for the pathways involved in the dysregulated redox-associated genes. (c) A bubble chart for the involved GO terms of the dysregulated redox-associated genes. (d) A bubble chart for the involved pathways of the dysregulated redox-associated genes. GO = Gene Ontology.

The dysregulated redox-associated genes participate in the response to oxidative stress, NADP activity, hormone metabolism, DNA packaging, and oxygen binding (Figures 3(a) and 3(c)). The dysregulated genes are mainly involved in alcoholism, neutrophil extracellular trap formation, systemic lupus erythematosus, IL-17 signaling pathway, and transcriptional misregulation in cancer (Figures 3(b) and 3(d)). The response to oxidative stress is the most significantly activated function of these dysregulated redox-associated genes, with almost 20% of the dysregulated redox-associated genes participating in this function.

### 3.3. Establishment of a Prognostic Model

**Figure 4:**
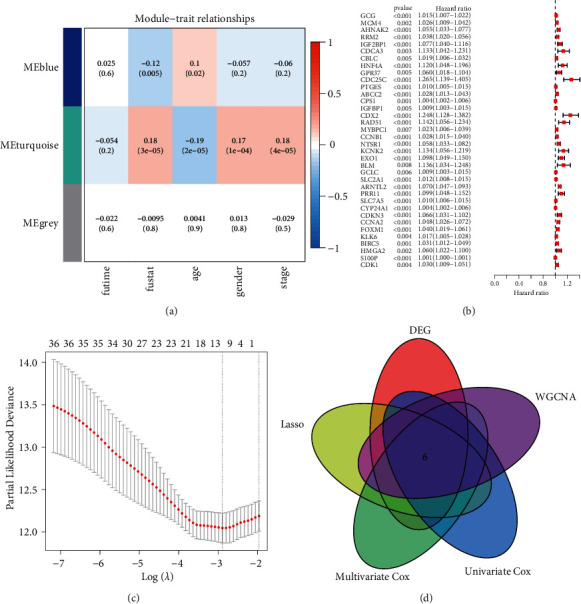
Construction of a prognostic model. (a) Three modules associated with clinical variables in WGCNA. (b) Forest graph of 36 prognostic redox-associated genes obtained by univariate Cox analysis. (c) Ten candidate prognostic genes identified by the LASSO Cox regression analysis. (d) Venn diagram of overlapping prognostic genes in WGCNA, univariate Cox analysis, LASSO Cox regression analysis, and multivariate Cox regression analysis. WGCNA = weighted correlation network analysis; LASSO = shrinkage and selection operator.

Two gene modules (MEblue and MEturquoise) were found to correlate with the survival of LUAD patients in WGCNA (Figure 4(a)). The MEturquoise module was selected for further analyses since it correlated with not only survival but also clinical parameters, including stage, age, and gender. Then, log-rank test and univariate Cox regression analysis identified 36 prognostic genes from 145 redox-associated genes in the MEturquoise module (Figure 4(b)). Ten prognostic genes (AHNAK2, IGF2BP1, CDC25 C, ABCC2, CPS1, CDX2, NTSR1, SLC2A1, ARNTL2, and SLC7A5) were identified by the LASSO regression (Figure 4(c)). Next, to avoid underestimation of coefficients and minimize variables, the multivariate Cox regression was performed to select 6 prognostic genes (AHNAK2, CDC25 C, CPS1, CDX2, NTSR1, and SLC2A1) to construct a prognostic model (Figure 4(d), Supplementary Table 1). All six prognostic genes were oncogenes adversely affecting survival and were statistically significant in the multivariate Cox regression analyses except *NTSR1*. The risk score can be calculated by adding together the products of the expression level of genes and corresponding coefficients for each patient (Supplementary Table 1). Patients in the TCGA and GEO cohorts were classified into a high-risk group and a low-risk group by the cutoff value of the median risk score. The clinical characteristics of the discovery cohort and validation cohort are shown in Table 1 and Supplementary Table 2.

### 3.4. The Prognostic Model Had Robust Prognostic Performance

Six prognostic redox-associated genes were differentially expressed in GSE36823 (Supplementary Figure 1(a)) and GSE43458 (Supplementary Figure 1(b)). The results of survival curves in GEPIA were also consistent with our findings. *AHNAK2* (HR = 1.5, *p* value = 0.0088), *CDC25 C* (HR = 2.5, *p* value < 0.001), *CPS1* (HR = 1.5, *p* value = 0.052), CDX2 (HR = 1.6, *p* value = 0.011), *NTSR1* (HR = 1.6, *p* value = 0.0035), and SLC2A1 (HR = 1.6, *p* value < 0.001) are unfavorable indicators of survival (Supplementary Figure 2(a)–(f)). In addition, *AHNAK2* (HR = 1.7, *p* value = 0.017), *CDC25 C* (HR = 1.9, *p* value = 0.0039), *CPS1* (HR = 1.6, *p* value = 0.028), *NTSR1* (HR = 1.7, *p* value = 0.038), and SLC2A1 (HR = 1.6, *p* value = 0.028) adversely affected disease-free survival (DFS) (Supplementary Figures 2(g), (h), (i), (k), and (l)), whereas no obvious difference in DFS was observed between the low-*CDX2* and high-*CDX2* groups (Supplementary Figure 2(j)).

**Figure 5:**
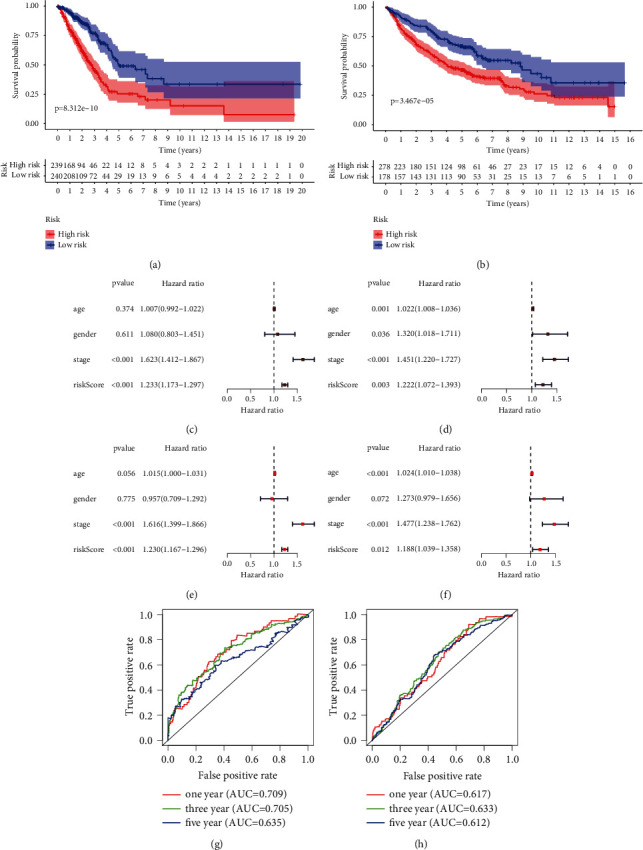
Validation of the prognostic model in the TCGA and GEO cohorts. (a) Survival curves of the low- and high-risk groups in the TCGA cohort. (b) Survival curves of the low- and high-risk groups in the GEO cohort. (c) Univariate Cox analysis of risk score and clinical variables in the TCGA cohort. (d) Univariate Cox analysis of risk score and clinical variables in the GEO cohort. (e) Multivariate Cox analysis of risk score and clinical variables in the TCGA cohort. (f) Multivariate Cox analysis of risk score and clinical variables in the GEO cohort. (g) ROC curves of the prognostic model for predicting 1‐, 3‐, and 5‐year survival rates in the TCGA cohort. (h) ROC curves of the prognostic model for predicting 1‐, 3‐, and 5‐year survival rates in the GEO cohort. TCGA = The Cancer Genome Atlas; GEO = Gene Expression Omnibus.

After verifying the differential expression and prognostic value of the genes in the model, the performance of the prognostic model was confirmed in the discovery (TCGA) and validation cohorts (GSE37745, GSE31210, and GSE50081). By survival curves and univariate Cox regression analysis, we identified the risk score as an unfavorable indicator of survival in both the TCGA (HR = 1.233, *p* value < 0.001) (Figures 5(a) and 5(c)) and GEO cohorts (HR = 1.222, *p* value = 0.003) (Figures 5(b) and 5(d)). The results of multivariate Cox regression analysis demonstrated that the prognostic value of our model was not affected by clinicopathological parameters, including age, gender, and stage, in the TCGA (HR = 1.23, *p* value < 0.001) (Figure 5(e)) and GEO cohorts (HR = 1.188, *p* value = 0.012) (Figure 5(f)). The AUCs for 1-, 3-, and 5-year survival in the TCGA cohort (Figure 5(g)) were 0.709, 0.705, and 0.635, respectively. The AUCs for 1-, 3-, and 5-year survival in the GEO cohort (Figure 5(h)) were 0.617, 0.633, and 0.612, respectively.

### 3.5. Risk Score Was Associated with Tumor Stage and Driver Gene Mutation

**Figure 6:**
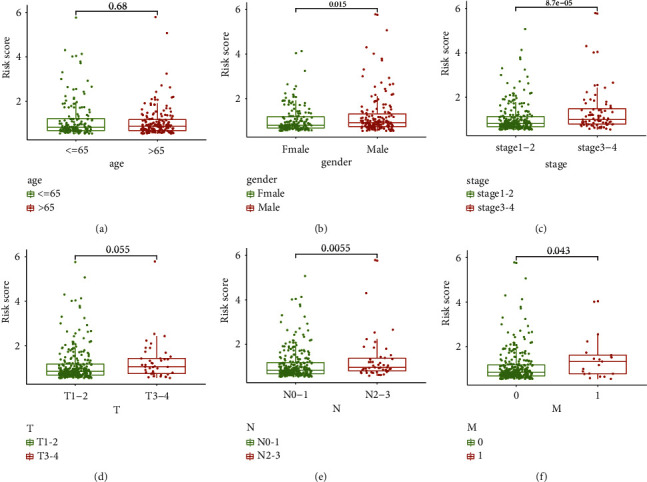
(a–f) Distribution of risk score between patients classified by clinical parameters (age, gender, stage, T stage, N stage, M stage).

**Figure 7:**
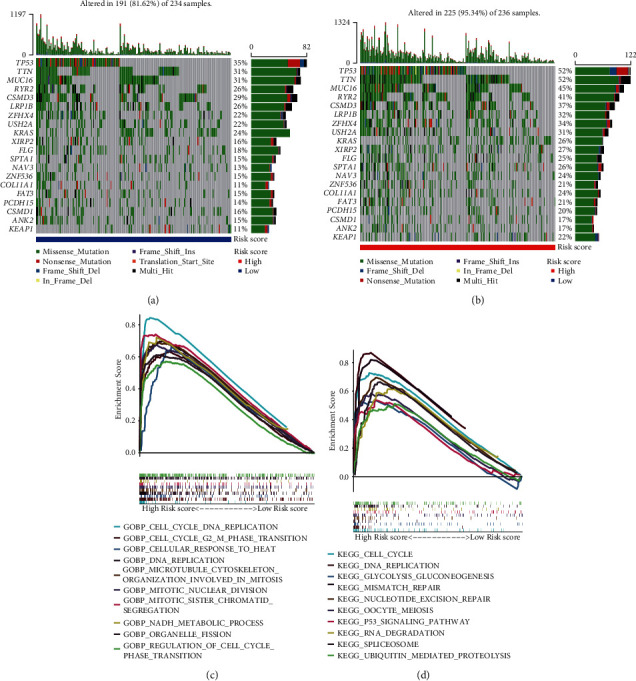
(a) Mutation status of genes with a high mutation rate in the low-risk group. (b) Mutation status of genes with a high mutation rate in the high-risk group. (c) Involved GO terms of the prognostic model. (d) Involved pathways of the prognostic model. GO = Gene Ontology.

A statistically significant difference in the distribution of risk score between patients stratified by clinicopathological status was observed. Although no association was observed between age and risk score (Figure 6(a)), the risk score was associated with gender, stage, lymph node metastasis, and distant metastasis (Figures 6(b)–6(f)). The risk score was higher in male patients and patients with advanced tumor, suggesting that the prognostic model was related to tumor progression and clinical outcome. Based on analysis of mutation data in TCGA, we found that the low-risk group and the high-risk group had significantly different gene mutation frequencies (Figures 7(a) and 7(b)). The low-risk group had a higher rate of mutated *EGFR* (15.38% vs 8.9%, *p* value = 0.031) and a lower rate of mutated TP53 (35.04% vs 51.69%, *p* value < 0.001) than the high-risk group (Table 2). Gene set enrichment analysis revealed that the prognostic model was mainly involved in the regulation of the cell cycle, DNA replication and repair, NADH metabolism, and the p53 signaling pathway (Figures 7(c)and 7(d)).

### 3.6. The Prognostic Model Was Related to Infiltration of Lymphocytes

**Figure 8:**
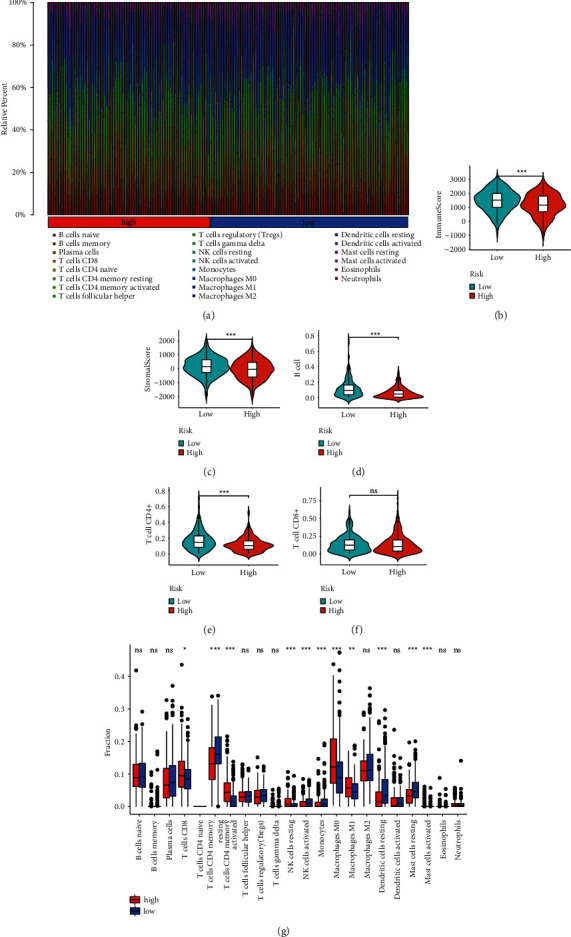
Correlation of the prognostic model with immunophenotypes. (a) Composition of immune cells of each sample in the low- and high-risk groups. (b–f) Immune score, stromal score, B cells, CD4+ T cells, and CD8+ T cells between the low- and high-risk groups. (g) Proportion of immune cells between the low- and high-risk groups.

We analyzed the infiltration of lymphocytes to gain an insight into the immune implications of the prognostic model. The immune score and stromal score were higher in the low-risk group than that in the high-risk group (*p* < 0.001) (Figures 8(b) and 8(c)). The abundance of immune cells such as B cells, CD4^+^ T cells, dendritic cells, and mast cells was higher in the low-risk group than that in the high-risk group (*p* < 0.001) (Figures 8(a), 8(d), 8(e), 8(f), and 8(g)). Thus, ICIs may achieve better therapeutic effects in the low-risk group since tumor-infiltrating immune cells are essential for the response to ICIs.

### 3.7. Established Nomogram Could Predict Survival Accurately

**Figure 9:**
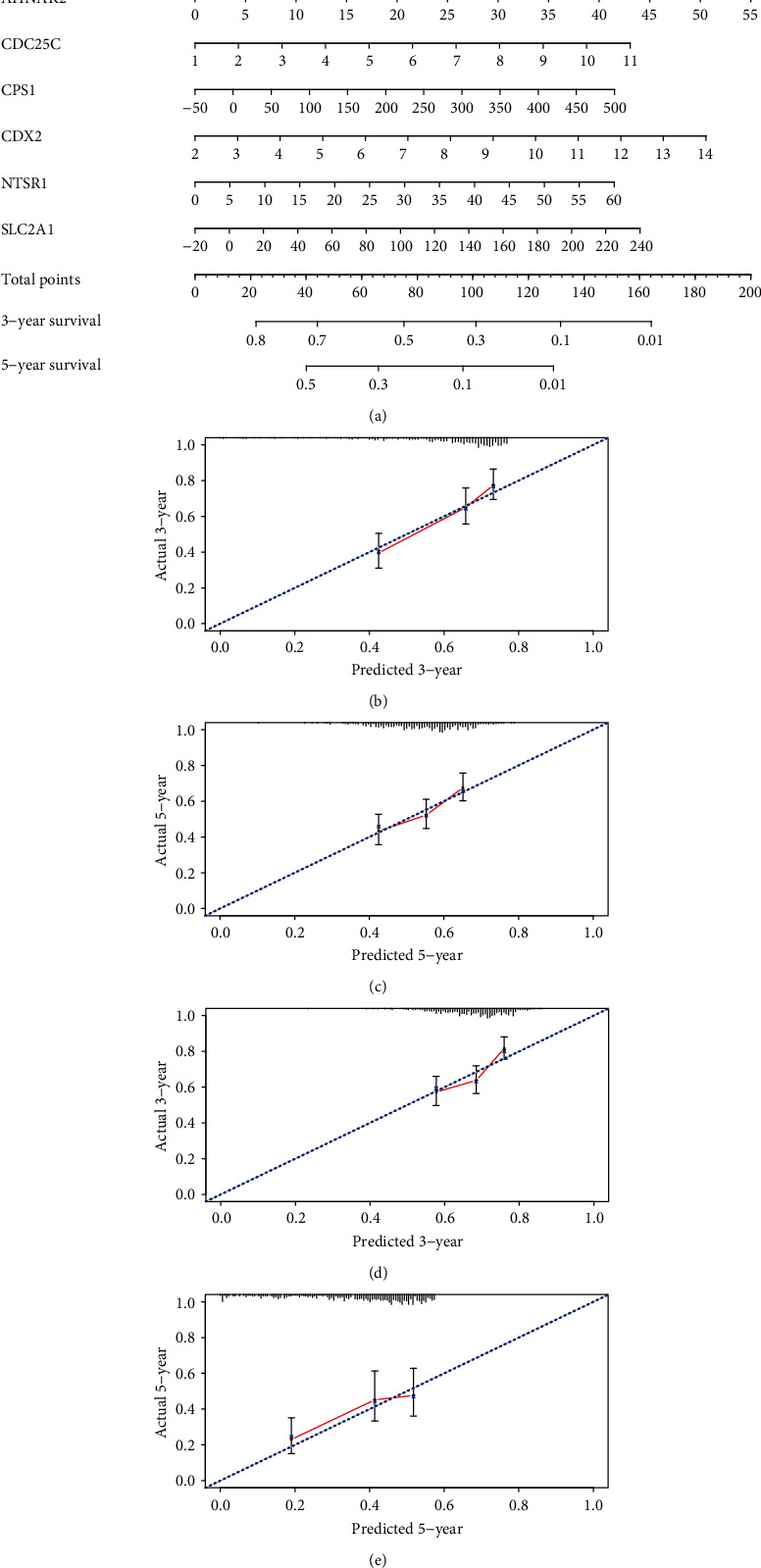
Visualization and verification of the prognostic model. (a) Nomogram based on the prognostic model for survival prediction. (b) Calibration curves showing the accuracy of the nomogram for predicting 3‐year survival rates in the TCGA cohort. (c) Calibration curves showing the accuracy of the nomogram for predicting 5‐year survival rates in the TCGA cohort. (d) Calibration curves showing the accuracy of the nomogram for predicting 3‐year survival rates in the GEO cohort. (e) Calibration curves showing the accuracy of the nomogram for predicting 5‐year survival rates in the GEO cohort. TCGA = The Cancer Genome Atlas; GEO = Gene Expression Omnibus.

Next, we established a nomogram to visualize the prognostic model for clinical application (Figure 9(a)). High prediction accuracy of the nomogram was reflected by calibration curves at 3 and 5 years in the TCGA (Figures 9(b) and 9(c)) and GEO (Figures 9(d) and 9(e)) cohorts.

## 4. Discussion

Patients with LUAD have poor prognosis because of the presence of distant metastases. Only 7% of LUAD patients with distant metastases can survive 5 years or longer [1]. The carcinogenesis mechanism of LUAD is complex and involves various biological processes, among which redox is a critical part. Redox can affect the function of proteins or lead to protein degradation by modifying the thiol side chain of protein cysteine residues. Even fine-tuning of redox homeostasis can incur changes in cellular functions such as proliferation, differentiation, and immune response [7, 30, 31]. Redox has been proven to be a significant contributing factor of oncogenesis. However, no study has explored the role of redox-associated genes in lung adenocarcinoma.

In this work, we conducted a systematic analysis to identify novel prognostic biomarkers of LUAD based on datasets in TCGA and GEO. First, 290 dysregulated redox-associated genes were identified and found to be related to oxidative stress, NADP activity, oxygen binding, DNA packaging, nucleosome assembly, and transcriptional misregulation in cancer. The functions of these genes reflected that redox-associated genes were involved in not only redox actions but also the regulation of DNA replication and transcription. Then, the stepwise analyses identified six prognostic redox-associated genes (AHNAK2, CDC25 C, CPS1, CDX2, NTSR1, and SLC2A1) and constructed a prognostic model. These six redox-associated genes are all oncogenes that could adversely affect survival. Among the six redox-associated genes, *NTSR1* did not reach statistical significance in the multivariate Cox regression analysis. The effect of *NTSR1* on survival may be dependent on other factors, such as downstream target genes or upstream regulatory genes. Although the expression of *NTSR1* is not independently associated with survival, it may represent a class of genes with similar effects on survival since it was selected as a significant prognostic gene in both LASSO and multivariate Cox regression analyses.

*AHNAK2* belongs to the AHNAK family and was reported to act as an oncogene in papillary thyroid carcinoma, pancreatic ductal adenocarcinoma, and clear cell renal cell carcinoma [32–34]. *AHNAK2* was found to facilitate invasion and migration in uveal melanoma and LUAD [35, 36]. Additionally, *AHNAK2* was correlated with immune infiltration in LUAD and papillary thyroid cancer [37, 38]. *CDC25 C* encodes the cell division cycle 25 C protein, serving as an activator of cyclin-dependent kinase 1, which regulates G2/M cell cycle transition. *CDC25 C* is essential to the regulation of cell cycle and DNA damage repair. The overexpression of CDC25 C aided in the evasion of cell death in nonmelanoma skin cancer [39]. *CDC25 C* is upregulated in many tumors and associated with unfavorable outcome [40–44]. CPS1 encodes carbamoyl phosphate synthetase 1, a key enzyme regulating the urea cycle. The inhibition of *CPS1* in lung cancer cells carrying KRAS/LKB1 mutations suppressed tumor growth [45]. The downregulation of *CPS1* was related to unfavorable survival in hepatocellular carcinoma [46]. Consistent with our findings, CPS1 was identified as an oncogene in LUAD, and the knockdown of *CPS1* could suppress cell growth and enhance the efficacy of gemcitabine [47]. Interestingly, evidence has shown that p53 can downregulate *CPS1* to inhibit tumor growth [48]. Studies have shown that caudal type homeobox 2 (*CDX2*) acts as a tumor suppressor gene to inhibit malignant phenotypes of colorectal cancer and that the low expression of *CDX2* is correlated with poor survival [49–52]. Wang et al. found that *CDX2* was able to activate natural killer cells to enhance the immune response in head and neck squamous cell carcinoma by upregulating CXCL14 [53]. Neurotensin receptor 1 (*NTSR1*) can combine with neurotensin (NTS) to form a complex to promote tumor progression in solid tumors such as prostate cancer, colorectal cancer, and pancreatic cancer [54–56]. The overexpression of *NTSR1* has been shown to indicate poor survival of patients with hepatocellular carcinoma and result in the activation of *EGFR* [57]. *SLC2A1* encodes GLUT-1 protein, which is an important glucose transporter in glucose metabolism. Evidence suggests that GLUT-1 is upregulated in prostate cancer and that the knockdown of GLUT-1 leads to proliferation inhibition and cell cycle arrest of prostate cancer cells [58]. *SLC2A1* was identified as an adverse prognostic factor of gastric cancer [59]. A recent study also demonstrated that *SLC2A1* was overexpressed in LUAD and was associated with shorter survival of patients, which was consistent with our findings [60,61].

Then, a series of analyses were conducted to verify the prognostic performance of the model. First, the differential expression of the prognostic genes in the model was validated in GSM43458 and GSM32863. Then, the prognostic value of these genes in the model was validated in GEPIA, and the results confirmed that all of the genes were oncogenes adversely affecting survival. Next, the robust prognostic performance of the model was demonstrated in the TCGA and GEO cohorts (GSE37745, GSE31210, and GSE50081). The results of the Kaplan–Meier survival curves and the univariate Cox regression analysis in both the TCGA and GEO cohorts showed that patients with low-risk score had a better prognosis. The multivariate Cox analysis further indicated that the risk score based on our model was an independent risk factor for survival. The AUCs for 1, 3, and 5 years in the TCGA and GEO cohorts suggested that the prognostic model possesses robust predictive power.

We also evaluated the relationship of the prognostic model with clinicopathological variables. The low-risk score was associated with early stage, low mutation rate of *TP53*, and high mutation rate of *EGFR*, which supported our findings that the patients with low-risk score had favorable survival. *TP53* encodes p53, which is a common tumor suppressor protein. The mutation of *TP53* can lead to loss of its function in tumor suppression and was proven to be correlated with unfavorable prognosis in lung cancer [62, 63]. A clinical trial suggested that the mutation of *TP53* indicated poor survival in lung cancer patients receiving chemotherapy after surgery [64]. Patients with mutations in *EGFR* are more likely to respond to tyrosine kinase inhibitors. GSEA revealed that the prognostic model was mainly involved in the regulation of cell cycle, DNA replication and repair, NADH metabolism, and p53 signaling pathway, which are vital processes affecting the proliferation of cancer cells and tumor progression.

Since redox can affect the function of lymphocytes and immune response [7, 65], we speculated that the prognostic model was associated with antitumor immunity. We evaluated the correlation between the prognostic model and immunophenotypes to find out that the low-risk score was associated with high infiltration of lymphocytes such as B cells, CD4^+^ T cells, and dendritic cells. The abundance of tumor-infiltrating lymphocytes was positively related to the efficacy of immune checkpoint inhibitors [66, 67]. B cells produce immunoglobulins and regulate T cells to enhance the antitumor response [68]. CD4^+^ T cells can increase immune response by activating CD8^+^ T cells [69, 70]. The antigen-presenting function of dendritic cells is essential for the activation of T cells. These results suggested that patients with low-risk score may have stronger antitumor immunity, and immunotherapy may work better in these patients than in those with high-risk score. Our model can be a potential tool to identify proper candidates for immunotherapy.

Limitations of our study are listed as follows. First, since our study was a retrospective study based on the analyses of data in public databases, the prognostic performance of the model constructed in our study needs to be tested in a prospective cohort study. Second, mechanistic exploration was not performed in our study, which needs to be conducted by in vivo and in vitro studies to explain the exact role of prognostic redox-associated genes in tumorigenesis and tumor immunity.

## 5. Conclusions

In conclusion, we introduced a novel redox-based prognostic model, which had robust prognostic performance in LUAD. The prognostic model can be a potential theranostic indicator that aids in predicting the survival of patients and identifying proper candidates for immunotherapy. Our findings explored novel prognostic biomarkers and contributed to tailoring optimal treatment strategies for patients with LUAD.

## Figures and Tables

**Table 1:** Clinicopathological variables of the discovery cohort and validation cohort.

Characteristics	Discovery cohort (*N* = 479)	Validation cohort (*N* = 456)
Age (years), *n* (%)		
<65	213 (44.47)	210 (46.05)
≥65	266 (55.53)	246 (53.95)
Gender, *n* (%)		
Female	260 (54.28)	208 (45.61)
Male	219 (45.72)	248 (54.39)
Stage, *n* (%)		
I	259 (54.07)	308 (67.54)
II	117 (24.43)	117 (25.66)
III	78 (16.28)	27 (5.92)
IV	25 (5.22)	4 (0.88)
Survival status, *n* (%)		
Dead	177 (36.95)	238 (52.19)
Alive	302 (63.05)	218 (47.81)

**Table 2:** Comparison of the mutation rates of TP53 and EGFR between the low- and high-risk groups by the chi-square test.

Variables	High risk	Low risk	*χ * ^2^	*p* Value
TP53				
Wild	114 (48.31%)	152 (64.96%)		
Mutant	122 (51.69%)	82 (35.04%)	13.263	<0.001
EGFR				
Wild	215 (91.1%)	198 (84.62%)		
Mutant	21 (8.9%)	36 (15.38%)	4.639	0.031

## Data Availability

The datasets analyzed in this study are available at TCGA (https://portal.gdc.cancer.gov/) and GEO (https://www. ncbi.nlm.nih.gov/geo).

## Abbreviations

abrLUAD: lung adenocarcinoma
TCGA: The Cancer Genome Atlas
WGCNA: weighted correlation network analysis
LASSO: least absolute shrinkage and selection operator
ROC curve: receiver operating characteristic curve
GSEA: gene set enrichment analysis
ICI: immune checkpoint inhibitor
ROS: reactive oxygen species
NOXs: NADPH oxidases
GEO: Gene Expression Omnibus
FDR: false discovery rate
GO: Gene Ontology
KEGG: Kyoto Encyclopedia of Genes and Genomes
GEPIA: Gene Expression Profiling Interactive Analysis
ESTIMATE: Estimation of STromal and Immune cells in Malignant Tumours using Expression data
CIBERSORT: Cell-type Identification By Estimating Relative Subsets Of RNA Transcripts
OS: overall survival
DFS: disease-free survival
HR: hazard ratio
CI: confidence interval

## Supplementary Materials

Supplementary Materials Supplementary Figure 1. Validation of 6 redox-associated genes in GEO. (a) Differential expression of 6 prognostic redox-associated genes in GSE32863. (b) Differential expression of 6 prognostic redox-associated genes in GSE43458. GEO = Gene Expression Omnibus. Supplementary Figure 2. Validation of 6 redox-associated genes in GEPIA. (A–F) Survival curves showing overall survival of patients with LUAD divided by expression of 6 redox-associated genes (*AHNAK2*, *CDC25 C*, *CPS1*, *CDX2*, *NTSR1*, and *SLC2A1*). (G–L) Survival curves showing the disease-free survival of patients with LUAD divided by expression of 6 redox-associated genes (*AHNAK2*, *CDC25 C*, *CPS1*, *CDX2*, *NTSR1*, and *SLC2A1*). GEPIA = Gene Expression Profiling Interactive Analysis. Supplementary Table 1: multivariate Cox regression analysis of prognostic redox-associated genes. Supplementary Table 2: clinical features of the discovery cohort.
