# Supplementary material for: Construction of a Redox-Related Prognostic Model with Predictive Value in Survival and Therapeutic Response for Patients with Lung Adenocarcinoma
Source: J Healthc Eng. 2022 Feb 25;2022:7651758. doi: 10.1155/2022/7651758 (PMC8896929; doi:10.1155/2022/7651758)
Supplement: Supplementary Materials — Supplementary Figure 1. Validation of 6 redox-associated genes in GEO. (a) Differential expression of 6 prognostic redox-associated genes in GSE32863. (b) Differential expression of 6 prognostic redox-associated genes in GSE43458. GEO = Gene Expression Omnibus. Supplementary Figure 2. Validation of 6 redox-associated genes in GEPIA. (A–F) Survival curves showing overall survival of patients with LUAD divided by expression of 6 redox-associated genes (AHNAK2, CDC25 C, CPS1, CDX2, NTSR1, and SLC2A1). (G–L) Survival curves showing the disease-free survival of patients with LUAD divided by expression of 6 redox-associated genes (AHNAK2, CDC25 C, CPS1, CDX2, NTSR1, and SLC2A1). GEPIA = Gene Expression Profiling Interactive Analysis. Supplementary Table 1: multivariate Cox regression analysis of prognostic redox-associated genes. Supplementary Table 2: clinical features of the discovery cohort. [file 7651758.f1.zip › 7651758.f1/Supplementary Table1.docx]

**Supplementary Table 1**: Multivariate Cox regression analysis of prognostic redox-associated genes.

| id | coef | HR | 95%CI | pvalue |
| --- | --- | --- | --- | --- |
| AHNAK2 | 0.0387 | 1.039 | 1.012-1.068 | 0.005 |
| CDC25C | 0.1666 | 1.181 | 1.043-1.339 | 0.009 |
| CPS1 | 0.0029 | 1.003 | 1.001-1.005 | 0.017 |
| CDX2 | 0.1617 | 1.176 | 1.045-1.322 | 0.007 |
| NTSR1 | 0.0268 | 1.027 | 0.999-1.056 | 0.061 |
| SLC2A1 | 0.0066 | 1.007 | 1.002-1.011 | 0.003 |

HR = hazard ratio, CI = confidence interval
