# Supplementary material for: Construction of a Redox-Related Prognostic Model with Predictive Value in Survival and Therapeutic Response for Patients with Lung Adenocarcinoma
Source: J Healthc Eng. 2022 Feb 25;2022:7651758. doi: 10.1155/2022/7651758 (PMC8896929; doi:10.1155/2022/7651758)
Supplement: Supplementary Materials — Supplementary Figure 1. Validation of 6 redox-associated genes in GEO. (a) Differential expression of 6 prognostic redox-associated genes in GSE32863. (b) Differential expression of 6 prognostic redox-associated genes in GSE43458. GEO = Gene Expression Omnibus. Supplementary Figure 2. Validation of 6 redox-associated genes in GEPIA. (A–F) Survival curves showing overall survival of patients with LUAD divided by expression of 6 redox-associated genes (AHNAK2, CDC25 C, CPS1, CDX2, NTSR1, and SLC2A1). (G–L) Survival curves showing the disease-free survival of patients with LUAD divided by expression of 6 redox-associated genes (AHNAK2, CDC25 C, CPS1, CDX2, NTSR1, and SLC2A1). GEPIA = Gene Expression Profiling Interactive Analysis. Supplementary Table 1: multivariate Cox regression analysis of prognostic redox-associated genes. Supplementary Table 2: clinical features of the discovery cohort. [file 7651758.f1.zip › 7651758.f1/Supplementary Table2.docx]

**Supplementary Table 2:** Clinical features of the discovery cohort.

| Characteristics | High-risk | Low-risk |
| --- | --- | --- |
|  | (N=239) | (N=240) |
| **Age** (years), n(%) |  |  |
| <65 | 108(45.19) | 105(43.75) |
| ≥65 | 131(54.81) | 135(56.25) |
| **Gende**r, n(%) |  |  |
| Female | 110(46.03) | 150(62.50) |
| Male | 129(53.97) | 90(37.50) |
| **Stage**, n(%) |  |  |
| I | 100(41.84) | 159(66.25) |
| II | 71(29.71) | 46(19.17) |
| III | 52(21.76) | 26(10.83) |
| IV | 16(6.69) | 9(3.75) |
| **T stage**, n(%) |  |  |
| T1 | 64(26.78) | 100(41.67) |
| T2 | 137(57.32) | 114(47.50) |
| T3 | 27(11.3) | 17(7.08) |
| T4 | 10(4.18) | 7(2.92) |
| Tx | 1(0.42) | 2(0.83) |
| **N stage**, n(%) |  |  |
| N0 | 135(56.49) | 176(73.33) |
| N1 | 55(23.01) | 35(14.58) |
| N2 | 45(18.83) | 22(9.17) |
| N3 | 2(0.84) | 0(0.00) |
| Nx | 2(0.84) | 7(2.92) |
| **M stage**, n(%) |  |  |
| M0 | 158(66.11) | 158(65.83) |
| M1 | 16(6.69) | 8(3.33) |
| Mx | 65(27.20) | 74(30.83) |
| **TP53**, n(%) |  |  |
| Wild | 114(47.70) | 152(63.33) |
| Mutant | 122(51.05) | 82(34.17) |
| Unkown | 3(1.26) | 6(2.50) |
| **EGFR**, n(%) |  |  |
| Wild | 215(89.96) | 198(82.5) |
| Mutant | 21(8.79) | 36(15.00) |
| Unkown | 3(1.26) | 6(2.50) |
