# Supplementary figures and images for: Construction of a Redox-Related Prognostic Model with Predictive Value in Survival and Therapeutic Response for Patients with Lung Adenocarcinoma
Source: J Healthc Eng. 2022 Feb 25;2022:7651758. doi: 10.1155/2022/7651758 (PMC8896929; doi:10.1155/2022/7651758)

**A**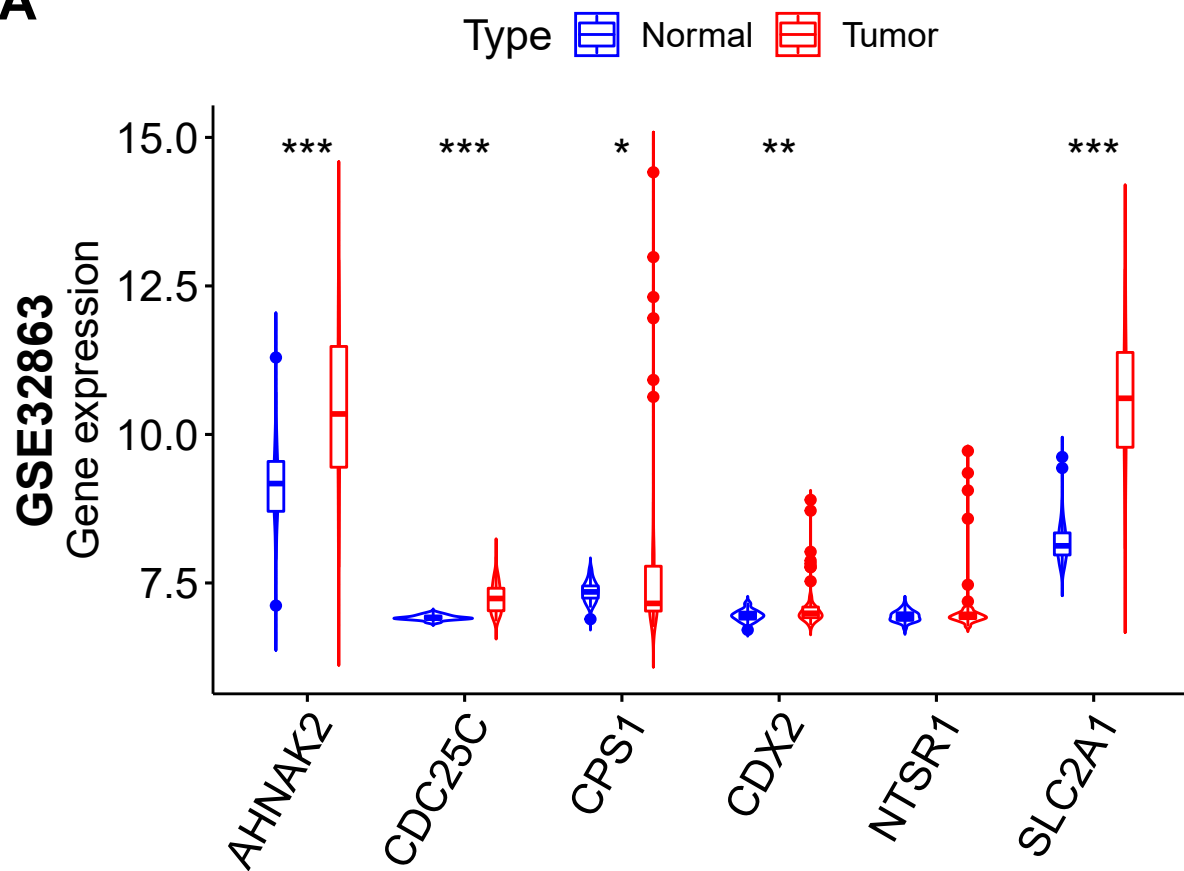**B**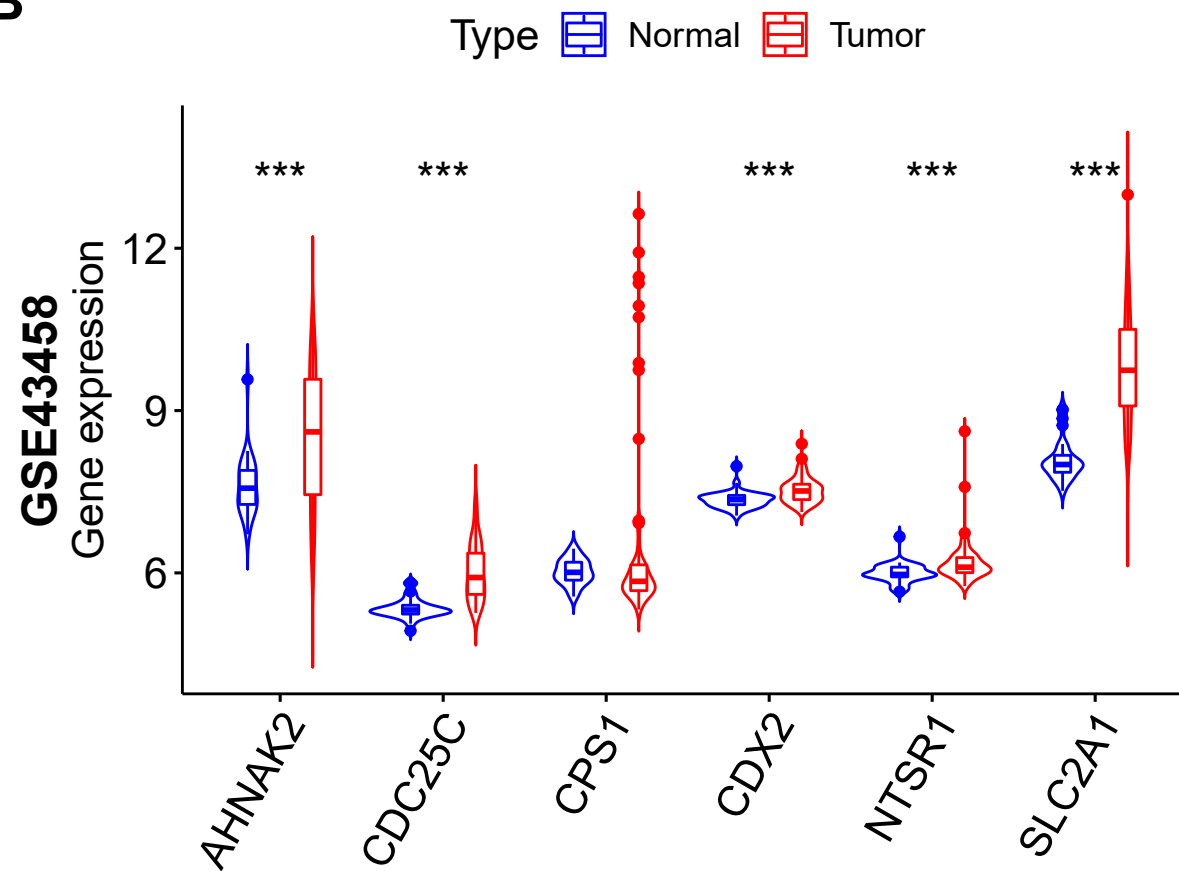

Supplement: Supplementary Materials — Supplementary Figure 1. Validation of 6 redox-associated genes in GEO. (a) Differential expression of 6 prognostic redox-associated genes in GSE32863. (b) Differential expression of 6 prognostic redox-associated genes in GSE43458. GEO = Gene Expression Omnibus. Supplementary Figure 2. Validation of 6 redox-associated genes in GEPIA. (A–F) Survival curves showing overall survival of patients with LUAD divided by expression of 6 redox-associated genes (AHNAK2, CDC25 C, CPS1, CDX2, NTSR1, and SLC2A1). (G–L) Survival curves showing the disease-free survival of patients with LUAD divided by expression of 6 redox-associated genes (AHNAK2, CDC25 C, CPS1, CDX2, NTSR1, and SLC2A1). GEPIA = Gene Expression Profiling Interactive Analysis. Supplementary Table 1: multivariate Cox regression analysis of prognostic redox-associated genes. Supplementary Table 2: clinical features of the discovery cohort. [file 7651758.f1.zip › 7651758.f1/Supplementary Figure 1.pdf]

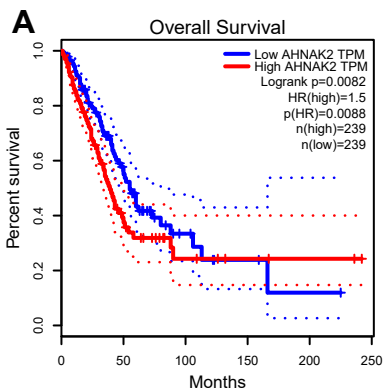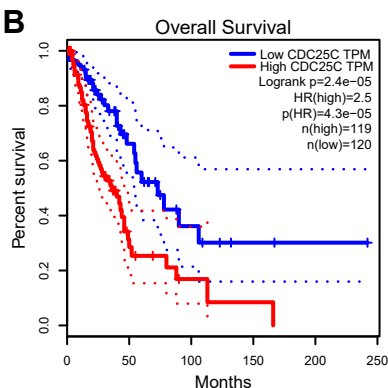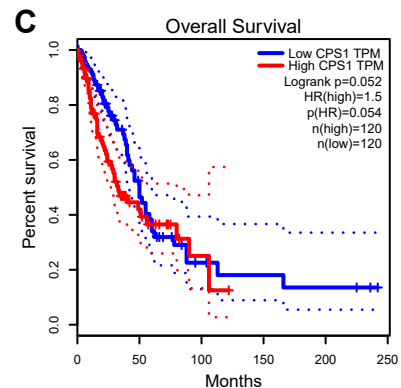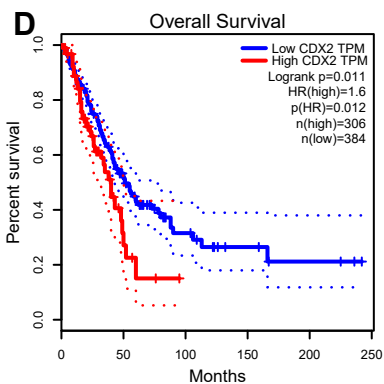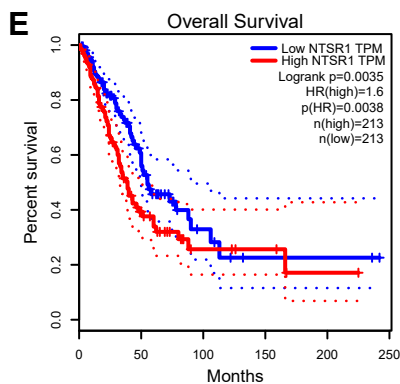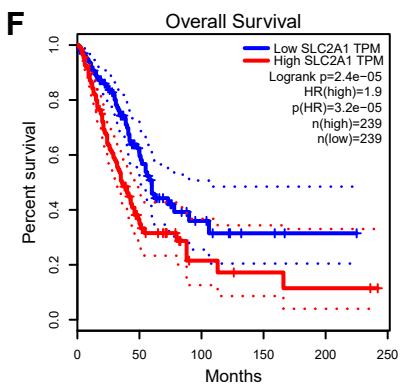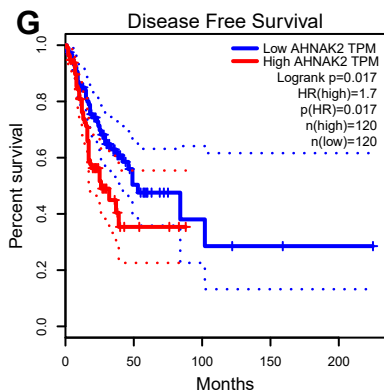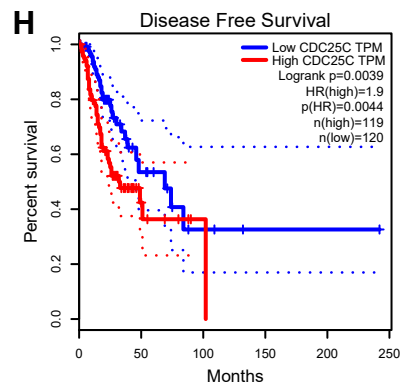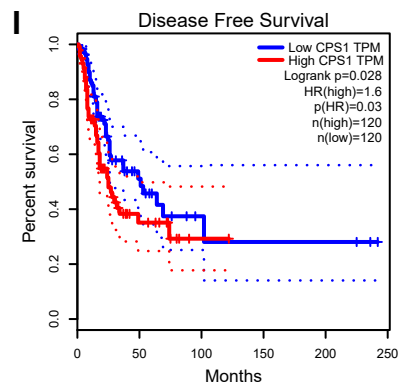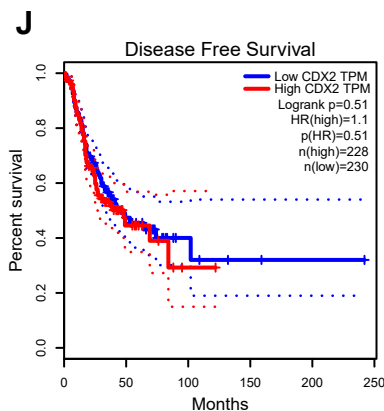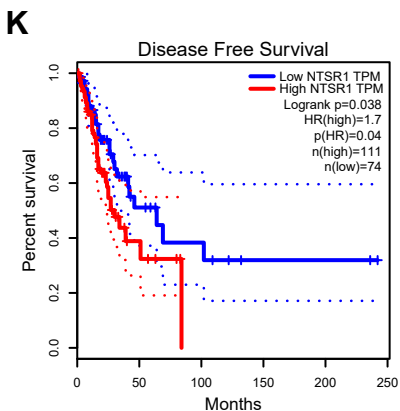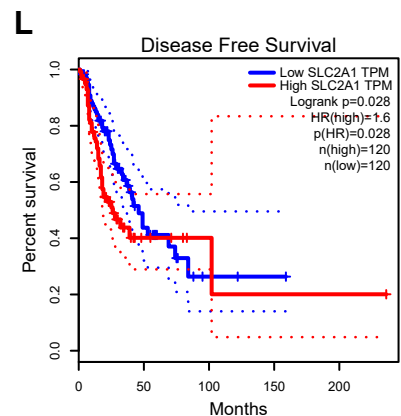

Supplement: Supplementary Materials — Supplementary Figure 1. Validation of 6 redox-associated genes in GEO. (a) Differential expression of 6 prognostic redox-associated genes in GSE32863. (b) Differential expression of 6 prognostic redox-associated genes in GSE43458. GEO = Gene Expression Omnibus. Supplementary Figure 2. Validation of 6 redox-associated genes in GEPIA. (A–F) Survival curves showing overall survival of patients with LUAD divided by expression of 6 redox-associated genes (AHNAK2, CDC25 C, CPS1, CDX2, NTSR1, and SLC2A1). (G–L) Survival curves showing the disease-free survival of patients with LUAD divided by expression of 6 redox-associated genes (AHNAK2, CDC25 C, CPS1, CDX2, NTSR1, and SLC2A1). GEPIA = Gene Expression Profiling Interactive Analysis. Supplementary Table 1: multivariate Cox regression analysis of prognostic redox-associated genes. Supplementary Table 2: clinical features of the discovery cohort. [file 7651758.f1.zip › 7651758.f1/Supplementary Figure2.pdf]
